# FRAX®: Prediction of Major Osteoporotic Fractures in Women from the General Population: The OPUS Study

**DOI:** 10.1371/journal.pone.0083436

**Published:** 2013-12-30

**Authors:** Karine Briot, Simon Paternotte, Sami Kolta, Richard Eastell, Dieter Felsenberg, David M. Reid, Claus-C. Glüer, Christian Roux

**Affiliations:** 1 Paris-Descartes University, Rheumatology Department, Cochin Hospital, Paris, France; 2 Department of Human Metabolism, University of Sheffield, Sheffield, United Kingdom; 3 Centre of Muscle and Bone Research, Charité – University Medicine Berlin, Campus Benjamin Franklin, Free and Humboldt University, Berlin, Germany; 4 School of Medicine & Dentistry, University of Aberdeen, Aberdeen, United Kingdom; 5 Biomedizinische Bildgebung, Klinik für Diagnostische Radiologie, Universitätsklinikum Schleswig-Holstein, Kiel, Germany; UCSD School of Medicine, United States of America

## Abstract

**Purposes:**

The aim of this study was to analyse how well FRAX® predicts the risk of major osteoporotic and vertebral fractures over 6 years in postmenopausal women from general population.

**Patients and methods:**

The OPUS study was conducted in European women aged above 55 years, recruited in 5 centers from random population samples and followed over 6 years. The population for this study consisted of 1748 women (mean age 74.2 years) with information on incident fractures. 742 (43.1%) had a prevalent fracture; 769 (44%) and 155 (8.9%) of them received an antiosteoporotic treatment before and during the study respectively. We compared FRAX® performance with and without bone mineral density (BMD) using receiver operator characteristic (ROC) c-statistical analysis with ORs and areas under receiver operating characteristics curves (AUCs) and net reclassification improvement (NRI).

**Results:**

85 (4.9%) patients had incident major fractures over 6 years. FRAX® with and without BMD predicted these fractures with an AUC of 0.66 and 0.62 respectively. The AUC were 0.60, 0.66, 0.69 for history of low trauma fracture alone, age and femoral neck (FN) BMD and combination of the 3 clinical risk factors, respectively. FRAX® with and without BMD predicted incident radiographic vertebral fracture (n = 65) with an AUC of 0.67 and 0.65 respectively. NRI analysis showed a significant improvement in risk assignment when BMD is added to FRAX®.

**Conclusions:**

This study shows that FRAX® with BMD and to a lesser extent also without FN BMD predict major osteoporotic and vertebral fractures in the general population.

## Introduction

Bone mineral density (BMD) measurement by dual x-ray absorptiometry (DXA) is regarded as the reference method for fracture prediction [1], [2]. However, BMD explains only a part of an individual's fracture risk because of the multiple determinants of fragility fracture [3]. Studies have shown that up to one half of patients with incident fractures have baseline BMD above the diagnostic threshold of osteoporosis T score ≤−2.5 [4], [5]. Thus attention must be paid to the identification of subjects at high risk of fracture, and many clinical risk factors predict the risk of fracture, independently of the BMD. The combination of BMD with risk factors can improve the detection of patients at high risk of osteoporotic fractures [6]–[12], including non vertebral [13]–[17], and vertebral fractures [17]–[23]. To identify persons at high risk for hip fracture and other fractures associated with osteoporosis, the WHO developed two 10-yr probabilities of fracture models (FRAX®): one for hip fracture and one for major osteoporotic fracture (hip, spine [clinical], wrist, or shoulder). FRAX® uses nine clinical risk factors to estimate the 10-yr probability of fracture: age, sex, body mass index, parental history of hip fracture, exposure to systemic glucocorticoïds, history of prior fragility fracture, current smoking, three or more units of alcohol per day, and the presence of secondary osteoporosis [24]. The validity of the WHO 10-yr probability of major osteoporotic fracture model (FRAX®) for prediction of fracture has been tested in some studies [24]–[30]. The National Osteoporosis Foundation recommendation is to use FRAX® only when the decision to treat or not to treat is difficult, i.e. mainly in postmenopausal women without osteoporosis and without prevalent fracture [31]. The Osteoporosis and Ultrasound Study (OPUS) is thus an appropriate population in which to assess the predictive value of FRAX®, as participants were postmenopausal women recruited from the general population, and thus were without referral bias. We analyzed how well FRAX® with and without femoral neck BMD (FN BMD), predicted the risk of incident major osteoporotic and vertebral fractures over 6 years in the OPUS cohort conducted in European postmenopausal women.

## Patients and Methods

### Patients

The Osteoporosis and Ultrasound Study (OPUS) is a multicenter prospective study of risk factors for fractures in post menopausal women. Both the rationale and the study design have been described in details elsewhere [32]. The initial study population consisted of 2409 ambulatory European women aged above 55 years, recruited in 5 European centers from random population samples between 1999 and 2001, and followed for 6 years. Women were excluded if they had disorders precluding ultrasound and bone mineral density measurements, and also general and cognitive inability that precluded completing questionnaire.

For this study, we included 1748 women with information on incident major osteoporotic fractures. The first outcome was the incident major osteoporotic fractures as defined by FRAX®. The secondary outcome was the incidence of radiographic incident vertebral fractures. We analysed the predictive value of FRAX® in the whole population and in a subgroup of 698 patients who had never been treated before or during the study, as it is recommended to use FRAX® only in untreated individuals [24].

### Ethics Statement

Human subjects review or ethics committees at each participating institution (“Ethikkommission der Medizinischen Fakultät der Christian-Albrechts-Universität zu Kiel” for Kiel, “Ethikkommission des Universitätsklinikum Benjamin Franklin der Freien Universität Berlin” for Berlin, “North Sheffield Research Ethics Committee” for Sheffield, “North of Scotland Research Ethics Committee, Grampian Health Board” for Aberdeen and “Comité consultatif de protection des personnes (CCPPRB) Paris-Cochin” for Paris) reviewed and approved the study. Written informed consent was obtained from all participants.

### Assessment of risk factors of FRAX® and calculation of FRAX® tool

All risk factors for fracture included in FRAX® were assessed baseline. Among the secondary osteoporosis categories in FRAX®, data on type 1 diabetes, osteogenesis imperfecta and chronic liver disease were not available. Prior non vertebral fractures were those that occurred after the age of 50 years, identified by self-reporting during the baseline questionnaire.

Bone mineral density of the lumbar spine and of the proximal femur was measured using dual energy X-ray absorptiometry (DXA) in the postero-anterior projection (Hologic QDR-4500; Hologic, Bedford, MA, USA in Paris, Kiel and Sheffield centers) or in the antero-posterior (Lunar Expert devices; GE Lunar, Madison, USA in the Berlin and Aberdeen centers) using standardised procedures and centralised quality control.

FRAX® was calculated by the WHO collaborating centre for Metabolic Bone Diseases, University of Sheffield, UK, using country-specific data. We used the FRAX® estimate of major osteoporotic fracture (including clinical spine fractures) in all analyses. Our model is conditional on the estimates of the 10-yr probability of fracture developed by FRAX®.

### Fracture assessment

Self-reports of incident fractures were confirmed by written reports of radiographs or surgical reports. We excluded pathologic fractures. A major osteoporotic fracture was defined as a fracture of the hip, spine (clinical), wrist, or humerus. Clinical vertebral fractures were defined as those that came to medical attention and were reported to the clinical centers by the participants. A copy of the radiograph obtained by the patient's physician was sent to the coordinating center and compared with the baseline study radiograph.

Vertebral fracture status was determined on lumbar and thoracic spine radiographs performed using a standardized procedure identical in all centers, and a standardized assessment in a central facility in the Berlin center. Radiographs were performed at baseline and final 6-year visits using the same procedures, and evaluated centrally by two radiologists. The procedure to assess fracture status combined morphometric measurements of vertebral heights and the qualitative interpretation of fracture status: vertebrae with deformities of non-osteoporotic origin (degenerative changes) or exhibiting potential misleading appearances were not considered as fracture. For both prevalent and incident deformities a decrease of at least 20% of any height or height ratio was considered for the diagnosis of fracture.

### Statistical analysis

The characteristics of women with and without incident major osteoporotic fractures were compared by using chi-square tests or Fisher's exact tests, or t-tests as appropriate.

We assessed the association between age, femoral neck (FN) BMD, history of low trauma fracture and combination of these factors. We tested the association of FRAX® with and without FN BMD. We used logistic regression to calculate the OR and 95% CI. We used the C-statistic and 95% CI to evaluate the discrimination of each model. The C-statistic estimates the area under the receiver operator characteristic (ROC) curve (AUC) and indicates the model's ability to distinguish those with and without incident major osteoporotic fractures and incident radiographic vertebral fractures. We analysed the predictive value of FRAX® in the whole population (n = 1748) and in the population who never received any anti-osteoporotic treatment (n = 698) and who ever received antiosteoporotic treatment (n = 1050).

Reassignment was evaluated by net reclassification improvement [33]–[34]. The NRI evaluates the movement of individuals between risk categories from one model to another. First, among those who fracture, the proportion moving upward from low-risk to high-risk category is subtracted from the proportion moving downward from high-risk to low-risk category. Second, among those who remain free of fracture, the proportion moving upward from low-risk to high-risk category is subtracted from the proportion moving downward from high-risk to low-risk category. Finally, 2 differences are summed; the higher the value, the more appropriate the reassignments. For this analysis, we determined 3 categories of risk using 2 thresholds. The first cut point was determined using the ROC threshold that gave the maximum Younden's Index [35]. (equal to the sensitivity plus the specificity minus 1) which corresponded to 3.5% 10-yr major osteoporotic fracture probability in the model with BMD. The second cut point was the clinical treatment threshold of 20% for major osteoporotic fractures proposed by the National Osteoporosis Foundation (NOF) [36].

Statistics were performed using Statistical Analysis Software (SAS V9.1, SAS Institute, Cary, NC, USA).

## Results

A group of 1748 women is the basis of this study, and their baseline characteristics are in the Table 1. Participants were followed from 4.5 years to 7.5 years (mean 6.04 years). 85 (4.9%) patients with incident major osteoporotic fractures were recorded including 9 patients with hip fractures; 65 (4.2%) patients with incident radiographic vertebral fractures were recorded. In patients with a history of low trauma fracture (n = 742, 43.1%), the incidence rate of major osteoporotic fracture and radiographic vertebral fractures were 6.7% and 5.0% respectively.

**Table:** Table 1. Baseline characteristics of the whole population with and without incident major osteoporotic fractures during the follow-up (n = 1748).

	Whole population	No incident fracture	Incident fracture	P value
**N (%)**	1748	1663 (95.1)	85 (4.9)	
**Age (years) (mean±SD)**	66.1 (6.8)	66.0 (6.7)	68.5 (7.4)	0.002
**Baseline lumbar spine T score, (mean ±SD)**	−0.95 (1.53)	−0.94 (1.54)	**−**1.33 (1.25)	0.055
**Baseline femoral neck T score, (mean±SD)**	−0.42 (1.05)	−0.41 (1.05)	**−**0.79 (0.98)	0.028
**Prevalent radiographic vertebral fractures N (%)**	219 (12.5)	197 (11.9)	22 (25.9)	0.0001
**FRAX clinical risk factors**				
History of low trauma fracture N (%)	742 (43.1)	692 (42.1)	50 (61.7)	0.001
BMI, (mean ±SD)	26.7 (4.5)	26.7 (4.5)	27.3 (3.9)	0.049
Parental history of hip fracture N, (%)	180 (10.5)	166 (10.2)	14 (18.2)	0.025
History of exposure to systemic glucocorticoids (%)	45 (2.7)	43 (2.7)	2 (2.5)	1.000
Rheumatoid arthritis (%)	108 (6.4)	102 (6.4)	6 (7.8)	0.631
Current smoking N (%)	236 (13.8)	222 (13.6)	14 (17.5)	0.328
Alcohol (≥ 3 units/day) N (%)	11 (0.6)	11 (0.7)	0 (0.0)	1.000
Secondary osteoporosis N (%)	521 (30.2)	500 (30.4)	21 (25.)	0.392
Type 1 diabetes,	_	_	_	_
Osteogenesis Imperfecta	_	_	_	_
Malnutrition	150 (9.8)	140 (9.6)	10 (13.9)	0.228
Malabsorption	7 (0.5)	7 (0.4)	0 (0)	1.000
Hypogonadism,	59 (3.8)	53 (3.6)	6 (8.2)	0.057
Menopause before 45 years	310 (20.0)	293 (19.8)	17 (23.3)	0.466
Untreated Hyperthyroidism	49 (3.3)	48 (3.4)	1 (1.4)	0.725
**FRAX 10-yr risk (%) of [mean (SD)]**				
Osteoporotic fracture, without BMD	13.03 (8.52)	12.88 (8.46)	16.18 (9.18)	0.001
Osteoporotic fracture, with BMD	10.23 (7.02)	10.05 (6.84)	14.38 (9.47)	<.0001
**Proportion of patients treated N (%)**				
Before and/or during the follow-up	1050 (60)	1000 (60.1)	50 (58.8)	0.810
During the study	155 (8.9)	136 (8.2)	19 (22.4)	<.0001

According to the inclusion criteria in OPUS, baseline mean T scores (±SD) were in the normal range at both the lumbar spine and the femoral neck (−0.95±1.53 and −0.42±1.05, respectively). The mean FRAX® value was 13.03 ± 8.52%.

### Prediction of the risk of incident major osteoporotic fractures in the whole population

Patients with incident major osteoporotic fractures were older, reported more frequently history of low trauma fracture and of prevalent radiographic vertebral fracture and had a significant lower baseline femoral neck BMD than patients without incident major osteoporotic fractures (Table 1).

**Figure 1:**
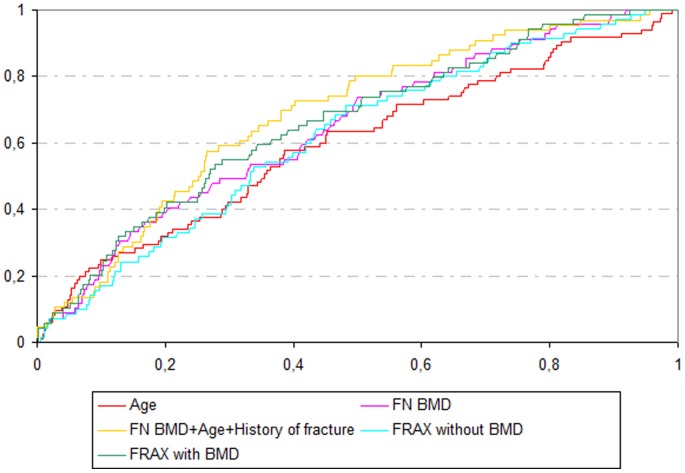
ROC curves for models to predict women with incident major osteoporotic fractures in the whole population.

In the table 1b are reported the baseline parameters associated with incident major osteoporotic fracture over 6 years. The AUCs are reported in table 2 and depicted in Figure 1. The highest AUC was observed for the combination of age, femoral neck BMD and previous history of fracture, which was significantly higher than the AUC for FRAX® without BMD (p≤0.0001) and for FRAX® with BMD (p = 0.021). The AUC for FRAX® with BMD was higher than the AUC for FRAX® without BMD (0.66 vs 0.62, p = 0.012). 8.3% of patients had a FRAX® with BMD ≥20% and 20.3% with incident fracture had a FRAX® with BMD ≥20%.

**Table 2:** Summary statistics for models to predict women with incident major osteoporotic fracture in the whole population.

Model	OR/SD (95%CI)	C-statistic (95%)
Age	1.43 [1.15 – 1.78]	0.60 [0.53 – 0.66]
FN BMD	1.80 [1.40 – 2.34]	0.65 [0.58 – 0.71]
History of low trauma fracture	1.48 [1.19 – 1.87]	0.60 [0.54 – 0.65]
Prevalent radiographic VF	1.37 [1.15 – 1.62]	0.57 [0.52 – 0.62]
FN BMD + age	1.57 [1.31 – 1.88]	0.66 [0.60 – 0.72]
FN BMD + age+ history of low trauma fracture	1.61 [1.34 – 1.92]	0.69 [0.63 – 0.75]
Baseline radiographic VF+ FN BMD + age	1.55 [1.31 – 1.81]	0.68 [0.61 – 0.74]
FRAX without FN BMD	1.37 [1.12 – 1.67]	0.62 [0.56 – 0.68]
FRAX with FN BMD	1.50 [1.26 – 1.77]	0.66 [0.60 – 0.73]

Using reassignment analysis, we examined the differences between FRAX® models estimates with and without BMD using 3 categories of risk 0<3.5%, 3.5–≤19%, and ≥20% (Table 3). We calculated the NRI to be 10.6% (p = 0.032), indicating a statistically significant improvement in risk assignment when BMD was added to the model. We also compared the differences between FRAX® model with BMD and the combination age, femoral neck BMD and previous history of fracture; the NRI was 2.4% (p = 0.711) indicating the absence of improvement risk assignment when the combination age, femoral neck BMD and previous was used.

**Table 3:** Fracture risk stratification, by calculated 10-year risks of major osteoporotic fracture from FRAX® models with and without BMD.

Model with BMD	Model without BMD			Total
Frequency (Row per cent)	< 3.5%	≥3.5 and ≤19%	≥ 20%	
*Patients with fracture*				
< 3.5%	13 (68.4%)	6 (31.6%)	0 (0.0%)	19
≥3.5 and ≤19%	2 (4.3%)	45 (95.7%)	0 (0.0%)	47
≥ 20%	0 (0.0%)	3 (100.0%)	0 (0.0%)	3
Total	15	54	0	69
*Patients without fracture*				
< 3.5%	522 (69.3%)	231 (30.7%)	0 (0.0%)	753
≥3.5 and ≤19%	44 (5.7%)	726 (94.3%)	0 (0.0%)	770
≥ 20%	0 (0.0%)	3 (75.0%)	1 (25.0%)	4
Total	566	960	1	1527

### Prediction of the risk of incident radiographic vertebral fractures in the whole population

Data were available in 1554 patients. Patients with incident vertebral radiographic fractures reported more frequently a history of prevalent radiographic vertebral fracture and of low trauma fracture, and had a significant lower baseline femoral neck BMD than patients without incident radiographic vertebral fractures (Table 4).

**Table 4:** Baseline characteristics of women in the whole population who had at least one radiographic vertebral fracture during follow-up and those who did not (n =  1554).

	No incident fracture	Incident fracture	P value
**N (%)**	1489 (95.8)	65 (4.2)	
**Age (years) (mean±SD)**	65.5 (6.6)	69.4 (6.7)	<0.0001
**Baseline lumbar spine T score (mean ±SD)**	−0.93 (1.52)	**−**1.43 (1.31)	0.039
**Baseline femoral neck T score (mean±SD)**	−0.38 (1.05)	**−**0.90 (1.01)	0.001
**Prevalent radiographic vertebral fractures N (%)**	164 (11.0)	27 (41.5)	<.0001
**FRAX clinical risk factors**			
History of low trauma fracture N (%)	613 (41.8%)	37 (57.8%)	0.011
BMI, (mean ±SD)	26.6 (4.4)	25.9 (4.3)	0.168
Parental history of hip fracture N, (%)	154 (10.6)	10 (15.6)	0.206
History of exposure to systemic glucocorticoïds (%)	32 (2.2)	1 (1.6)	1.000
Rheumatoid arthritis (%)	82 (5.7)	5 (7.9)	0.410
Current smoking N (%)	186 (12.8)	11 (17.2)	0.310
Alcohol (≥ 3 units/day) N (%)	8 (0.6)	1 (1.6)	0.322
Secondary osteoporosis N (%)	431 (29.4)	16 (25.0)	0.453
Type 1 diabetes,	_	_	_
Osteogenesis Imperfecta,	_	_	_
Malnutrition	139 (9.6)	10 (15.4)	0.125
Malabsorption	7 (0.5)	0 (0)	1.000
Hypogonadism,	56 (3.9)	2 (3.1)	1.000
Menopause before 45 years	296 (20.2)	12 (18.5)	0.732
Untreated Hyperthyroidism	45 (3.2)	1 (1.6)	1.000
**FRAX 10-yr risk (%) of [mean (SD)]**			
Osteoporotic fracture, without BMD	**12.3 (8.1)**	17.2 (11.3)	0.0001
Osteoporotic fracture, with BMD	**9.6 (6.7)**	14.7 (11.2)	<.0001
**Proportion of patients treated N (%)**			
In the past	907 (61.1)	46 (70.8)	0.112
At baseline and/over the follow-up	124 (8.3)	23 (35.4)	<.0001

**Figure 2:**
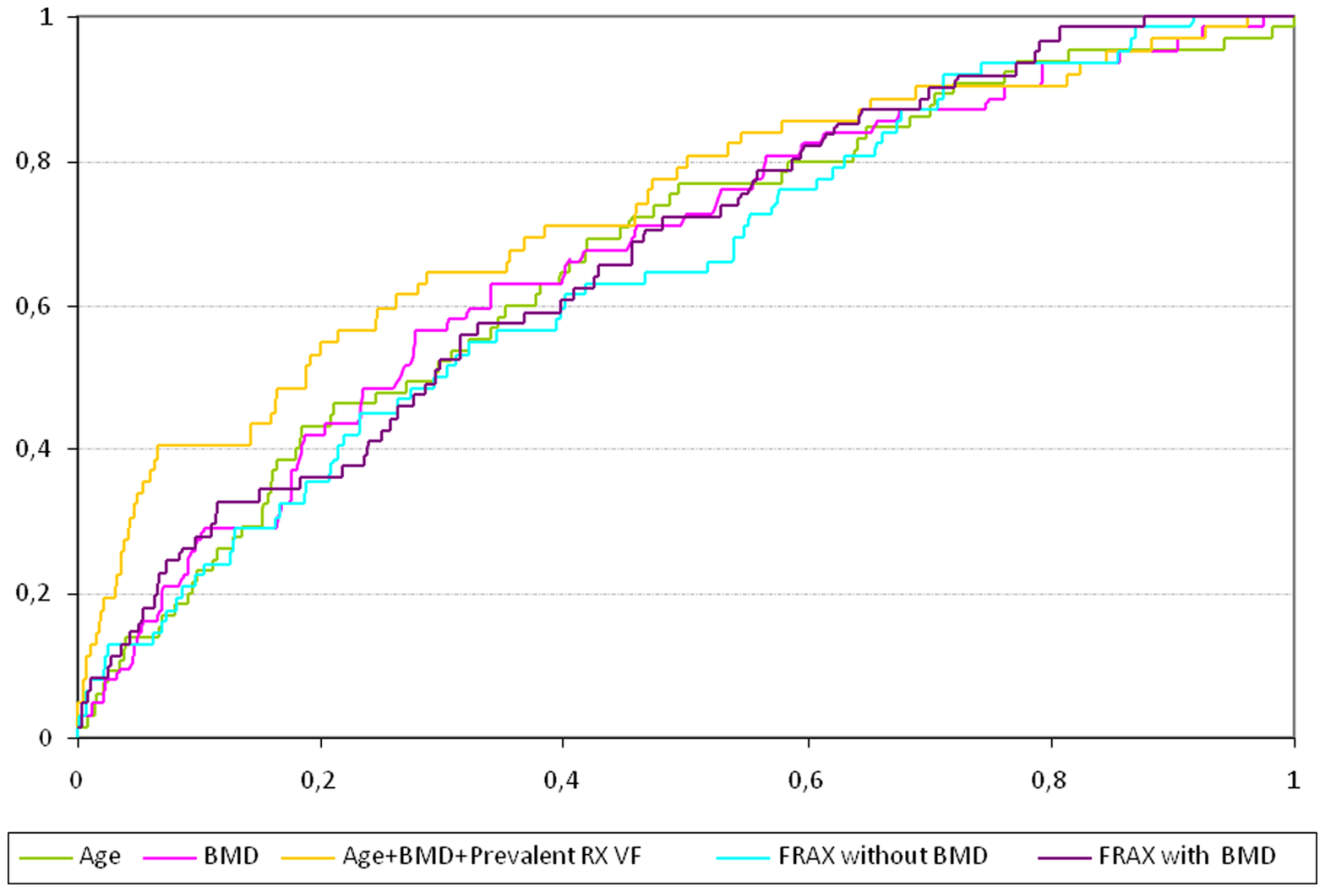
ROC curves for models to predict women with at Least One incident radiographic vertebral fracture.

In the table 2b are reported the baseline parameters associated with incident radiographic vertebral fracture over 6 years. The AUCs are reported in table 5 and in Figure 2. The highest AUC was observed for the combination of age, femoral neck BMD and baseline radiographic vertebral fracture, which was significantly higher than the AUC for FRAX® without BMD (p = 0.013) and for FRAX® with BMD (p = 0.005). The AUC for FRAX® with BMD was similar to the AUC for FRAX® without BMD (0.67 vs 0.65, p = 0.204).

**Table 5:** Summary statistics for models to predict women with at least one radiographically detected Vertebral Fracture.

Model	OR/SD (95%CI)	C-statistic (95%)
Age	1.77 [1.38 – 2.27]	0.66 [0.60 – 0.73]
FN BMD	1.87 [1.43 – 2.47]	0.67 [0.60 – 0.74]
History of low trauma fracture	1.38 [1.07 – 1.77]	0.58 [0.52 – 0.64]
Prevalent radiographic VF	1.78 [1.49 – 2.10]	0.65 [0.59 – 0.71]
FN BMD + age	1.64 [1.37 – 1.96]	0.69 [0.63 – 0.76]
FN BMD + age+ history of low trauma fracture	1.67 [1.39 – 1.99]	0.70 [0.63 – 0.77]
Baseline radiographic VF+ FN BMD + age	1.80 [1.56 – 2.09]	0.73 [0.66 – 0.80]
FRAX without FN BMD	1.57 [1.28 – 1.91]	0.65 [0.58 – 0.71]
FRAX with FN BMD	1.55 [1.30 – 1.84]	0.67 [0.60 – 0.73]

8.3% of patients had a MOF-FRAX® with BMD≥20% and 24.6% with incident radiographic vertebral fracture had a FRAX® with BMD≥20%. Reassignment analysis did not show significant improvement when BMD was added to FRAX without BMD or when the combination of age, femoral neck BMD and baseline radiographic vertebral fracture was used (data not shown).

### Prediction of the risk of incident major osteoporotic fracture in the subpopulation of postmenopausal women who never received any anti-osteoporotic treatment (n = 698)

Characteristics of those patients are summarized in Table 6 and were similar to those of the general population. In this subpopulation (n = 698), femoral neck BMD, history of low trauma fracture and the combination of age, femoral neck BMD and history of low trauma fractures were still predictors of incident major osteoporotic fractures OR  = 1.61 (1.08–2.40) (AUC =  0.58), OR  = 1.42 (1.09–1.81) (AUC =  0.65) and OR = 1.48 (1.00–2.13) (AUC =  0.70) respectively) (Table 7). FRAX® with and without BMD was not a predictor of incident major osteoporotic fractures OR =  1.33 (0.95–1.79) and OR =  1.27 (0.88–1.77) respectively.

**Table 6:** Baseline characteristics of women who never received any treatment with (n = 663) and without incident major osteoporotic fractures (35) during the follow-up (n =  698).

	Women untreated	No incident fracture	Incident fracture	P value
**N (%)**	698 (39.9)	663 (95.0)	35 (5.0)	_
**Age (years) (mean±SD)**	69.1 (6.4)	69.0 (6.3)	70.3 (8.1)	0.162
**Baseline lumbar spine T score (mean ±SD)**	−0.90 (1.44)	−0.89 (1.45)	−1.00 (1.35)	0.912
**Baseline femoral neck T score, (mean±SD)**	−0.42 (1.03)	−0.41 (1.03)	−0.76 (0.92)	0.272
**Prevalent radiographic vertebral fractures N (%)**	75 (10.7)	66 (10.0)	9 (25.7)	0.008
**FRAX clinical risk factors**				
History of low trauma fracture N (%)	286 (42.1)	263 (40.7)	23 (69.7)	0.001
BMI, (mean ±SD)	27.5 (4.6)	27.5 (4.7)	27.5 (3.9)	0.718
Parental history of hip fracture N, (%)	59 (8.8)	56 (8.7)	3 (9.4)	0.754
History of exposure to systemic glucocorticoids (%)	10 (1.5)	9 (1.4)	1 (3.2)	0.381
Rheumatoid arthritis (%)	48 (7.3)	43 (6.9)	5 (16.1)	0.067
Current smoking N (%)	80 (11.9)	76 (11.8)	4 (12.5)	0.784
Alcohol (≥ 3 units/day) N (%)	3 (0.4)	3 (0.5)	0 (0.0)	1.000
Secondary osteoporosis N (%)	214 (31.5)	207 (32.0)	7 (21.2)	0.249
Type 1 diabetes,	_	_	_	0.513
Osteogenesis Imperfecta,	_	_	_	_
Malnutrition,	57 (9.7)	53 (9.5)	4 (13.8)	
Malabsorption,	2 (0.3)	2 (0.4)	0 (0.0)	0.079
Hypogonadism,	21 (3.6)	18 (3.2)	3 (10.3)	-
Menopause before 45 years	119 (20.0)	116 (20.5)	3 (10.3)	0.184
Untreated Hyperthyroidism	17 (3.0)	16 (2.9)	1 (3.6)	0.577
**FRAX 10-yr risk (%) of [mean (SD)]**				
Osteoporotic fracture, without BMD	14.75 (8.21)	14.65 (8.12)	16.82 (9.85)	0.280
Osteoporotic fracture, with BMD	11.09 (6.16)	10.99 (6.08)	13.16 (7.44)	0.155

**Table 7:** Summary Statistics for Models to Predict Women with at Least One major osteoporotic fracture and who never received any antiosteoporotic.

Model	OR/SD (95%CI)	C-statistic (95%)
Age	1.23 [0.87 – 1.76]	0.57 [0.45 – 0.69]
FN BMD	1.61 [1.08 – 2.40]	0.58 [0.47 – 0.69]
History of low trauma fracture	1.42 [1.09 – 1.81]	0.65 [0.56 – 0.73]
Prevalent radiographic VF	1.04 [0.69 – 1.60]	0.58 [0.51 – 0.65]
FN BMD + age	1.35 [0.92 – 1.87]	0.59 [0.47 – 0.70]
FN BMD + age+ history of low trauma fracture	1.48 [1.00 – 2.13]	0.70 [0.61 – 0.78]
Baseline radiographic VF+ FN BMD + age	1.61 [1.21 – 2.10]	0.61 [0.50 – 0.73]
FRAX without FN BMD	1.27 [0.88 – 1.77]	0.56 [0.45 – 0.68]
FRAX with FN BMD	1.33 [0.95 – 1.79]	0.58 [0.47 – 0.70]

### Prediction of the risk of incident major osteoporotic fracture in the subpopulation of postmenopausal women who ever received any anti-osteoporotic treatment (n = 1050)

Characteristics of those patients are summarized in Table 8 and were similar to those of the general population. In this subpopulation (n = 1050), femoral neck BMD, history of low trauma fracture and the combination of age, femoral neck BMD and prevalent radiographic vertebral fracture were predictors of incident major osteoporotic fractures OR  = 1.59 (1.22 – 2.07) (AUC =  0.64), OR  = 1.95 (1.40 – 2.76) (AUC =  0.69) and OR = 2.33 (1.21 – 4.50) (AUC =  0.57) respectively) (Table 9). FRAX® with and without BMD were predictors of incident major osteoporotic fractures (OR = 1.43 (1.12 – 1.80) (AUC = 0.66) and (OR =  1.58 (1.28 – 1.94) (AUC = 0.70) without any difference.

**Table 8:** Baseline characteristics of women who ever received antiosteoporotic treatment t with (n = 50) and without incident major osteoporotic fractures (n = 1000) during the follow-up (n =  1050).

	Women treated	No incident fracture	Incident fracture	P value
**N (%)**	1050 (60.1)	1000 (95.2)	50 (4.8)	_
**Age (years) (mean±SD)**	64.1 (6.3)	64.0 (6.3)	67.2 (6.6)	0.001
**Baseline lumbar spine T score (mean ±SD)**	−0.99 (1.58)	−0.97 (1.59)	−1.57 (1.12)	0.015
**Baseline femoral neck T score (mean±SD)**	−0.83 (1.07)	−0.80 (1.07)	−1.57 (1.12)	<.0001
**Prevalent radiographic vertebral fractures N (%)**	144 (13.7)	131 (13.1)	13 (26.0)	0.010
**FRAX clinical risk factors**				
History of low trauma fracture N (%)	456 (43.7)	429 (43.1)	27 (56.3)	0.072
BMI, (mean ±SD)	26.2 (4.3)	26.1 (4.3)	27.2 (3.9)	0.028
Parental history of hip fracture N, (%)	121 (11.7)	110 (11.1)	11 (24.4)	0.007
History of exposure to systemic glucocorticoids (%)	35 (3.4)	34 (3.5)	1 (2.1)	1.000
Rheumatoid arthritis (%)	60 (5.9)	59 (6.1)	1 (2.2)	0.515
Current smoking N (%)	156 (15.1)	146 (14.8)	10 (20.8)	0.256
Alcohol (≥ 3 units/day) N (%)	8 (0.8)	8 (0.8)	0 (0.0)	1.000
Secondary osteoporosis N (%)	307 (29.4)	293 (29.4)	14 (29.2)	0.977
Type 1 diabetes,	_	_	_	_
Osteogenesis Imperfecta,	_	_	_	_
Malnutrition,	93 (9.8)	87 (9.6)	6 (14.0)	0.302
Malabsorption,	5 (0.5)	5 (0.6)	0 (0.0)	1.000
Hypogonadism,	38 (4.0)	35 (3.9)	3 (6.8)	0.414
Menopause before 45 years	191 (20.0)	177 (19.4)	14 (31.8)	0.044
Untreated Hyperthyroidism	32 (3.5)	32 (3.6)	0 (0.0)	0.394
**FRAX 10-yr risk (%) of [mean (SD)]**				
Osteoporotic fracture, without BMD	11.91 (8.53)	11.74 (8.48)	15.78 (8.83)	0.0003
Osteoporotic fracture, with BMD	9.68 (7.48)	9.43 (7.22)	15.16 (10.59)	<0.0001

**Table 9:** Summary Statistics for Models to Predict Women with at Least One major osteoporotic fracture and who received any antiosteoporotic treatment.

Model	OR/SD (95%CI)	C-statistic (95%)
Age	1.59 [1.22 – 2.07]	0.64 [0.57 – 0.72]
FN BMD	1.95 [1.40 – 2.76]	0.69 [0.61 – 0.76]
History of low trauma fracture	1.70 [0.95 – 3.05]	0.57 [0.49 – 0.64]
Prevalent radiographic VF	2.33 [1.21 – 4.50]	0.57 [0.50 – 0.63]
FN BMD + age	1.59 [1.27 – 1.98]	0.71 [0.63 – 0.79]
FN BMD + age+ history of low trauma fracture	1.60 [1.29 – 1.99]	0.71 [0.64 – 0.79]
Baseline radiographic VF+ FN BMD + age	1.55 [1.27 – 1.89]	0.72 [0.64 – 0.80]
FRAX without FN BMD	1.43 [1.12 – 1.80]	0.66 [0.59 – 0.74]
FRAX with FN BMD	1.58 [1.28 – 1.94]	0.70 [0.63 – 0.78]

### Discussion

This study shows that FRAX® with and without FN BMD can predict incident major osteoporotic over 6 years in European postmenopausal women recruited from general population. In this population which included proportion of women who had been treated by anti-osteoporotic treatment in past or who were treated during the follow-up, ROC c-statistical analysis showed that the performance of FRAX® with FN BMD was better than that of FRAX® without FN BMD. Because of the limitations of ROC analysis, reassignment analysis which has been proposed as a novel method to evaluate fracture risk models confirmed that the addition of BMD to FRAX® significantly increases the prediction, supporting results of previous studies [36]. Using ROC c-statistic analysis, the combination of FN BMD, age and self-reported fracture history significantly predicted incident major osteoporotic fractures with a higher predictive value than FRAX® with BMD. However, these results were not confirmed by the reassignment analysis.

In postmenopausal women who never received any anti-osteoporotic treatment, FRAX® with and without BMD was not a predictor of incident major osteoporotic fractures; however interpretation of the results is largely limited by the small number of women never treated. In women who ever received an anti-osteoporotic treatment, we confirmed that FRAX® with and without BMD can be used in patients currently or previously treated for osteoporosis [37]. However, FRAX® should not be used to assess the reduction in fracture risk in individuals on treatment.

The performance characteristics of the FRAX® tool have been validated in independent cohorts from various countries [24] with over a million of person-years of observation. Previous studies conducted in population with past/current osteoporotic treatment [30], [38], [39] and without such treatment [26], [40] reported the prediction of FRAX® in elderly women [30], [38], [39], [40] and in peri- and early postmenopausal women [26]. Using data of the SOF (Study of Osteoporotic Study) study, a cohort of older community-dwelling women (n =  6252, mean age of 71.3 years), Ensrud et al showed that a simple model based on age and BMD predicted 10-year risk of hip, major osteoporotic, and any clinical fracture as well as FRAX® models with BMD [30]. In the MENOS study (mean age 54±4 years) included 2,651 women (exclusion of women with past/current osteoporosis treatment> 3 months at baseline (n = 455) with a mean follow-up period of 13.4 years (±1.4 years), only a limited number of clinical risk factors were found to be associated with the risk of major OP fracture. In this population as in our study, the FRAX® did not significantly improve the discriminatory value of femur BMD alone [26].

The FRAX® tool is not designed to examine the risk of radiographic vertebral fractures; however we performed this exploratory analysis because of the importance of vertebral fractures in the outcome of osteoporosis, and because OPUS design gives the opportunity to assess that, as vertebral fractures were assessed on radiographs performed with standardized procedures of acquisition, by experts in a central facility, and without a context of clinical study conducted in an osteoporotic population. In this cohort of postmenopausal women (mean age 65.5 years), of whom 12.5% had a radiographically detected vertebral fracture at baseline and a mean lumbar spine of −0.95, FRAX® with and without BMD discriminate patients with incident radiographic vertebral fractures. Our study shows that the strongest risk factor of future vertebral fracture was the combination of age, femoral neck BMD and the presence of a radiographic vertebral fracture at baseline. There is only one previous study which analyses the predictive value of FRAX® for incident radiographic vertebral fractures. It was conducted in 3321 post-menopausal women with low bone mass (60% of them having a femoral neck T score ≤−2.5) from the FIT (Fracture Intervention Trial) placebo group, of whom 30% had a radiographically detected vertebral fracture at baseline. FRAX® with and without BMD did predict vertebral fracture [27]. These results of study are confirmed by our exploratory analysis conducted in a population with a lower prevalence of vertebral fractures at baseline, and a low prevalence of osteoporosis.

FRAX® has been included as a tool for identifying postmenopausal women in recently updated guidelines published by the NOF in the United States [31] and by the National Osteoporosis Guideline Group [NOGG]), in the UK [24], [41]. The NOF recommends using FRAX when the decision to treat or not to treat is uncertain. It is primarily intended for postmenopausal women and men 40 years of age and older who have T-scores between −1.0 SD and −2.5 SD and who are not on treatment, and who have not had spine or hip fractures [31]. In this general population, our study shows that the performance of FRAX® is mainly determined by the femoral neck BMD and previous fragility fracture, confirming previous studies [27], [30]. This lack of accuracy of FRAX® can be explained by some limitations of this algorithm, especially the disregard of some well established risk factors. However these limitations can be applied to all the prediction tools (Garvan fracture risk calculator, QF fracture) [42], [43]. The task forces of the ISCD (International Osteoporosis Foundation) and of the IOF (International Osteoporosis Foundation) recently reviewed and suggested explanations for the limitations of FRAX®, and particularly the risk factors not considered [44]. Falls and risk factors for falls are excluded from the FRAX® because of the lack of standardized evaluation methods and the lack of fracture prevention data with fall prevention measures. Additional risk factors for fractures, such as the number of causes of secondary osteoporosis, were not included because of the weak evidence that they increase the risk of fracture independently of BMD. Biochemical markers of bone turnover were not in FRAX® because of the biological variability, the lack of reference analytes and analytical standards [44]. An attempt to adjust the result of FRAX® based on dose of corticosteroid has been recently published [45].

Our study has several limitations. The first limitation concerns the characteristics of the cohort: low rate of incident fracture, quite short duration (6 years) of the follow-up and the significant proportion of women previously or currently receiving bone protective therapy which limit a lot the interpretation of the results. Our data has been obtained in a population including patients previously treated with anti-osteoporotic treatments. We showed that FRAX® is a predictor of major osteoporotic fracture in patients ever treated and not in patients never treated. We could not confirm that the performance of FRAX® is different or not in untreated individuals, because our sample size of untreated subjects was too low. Other statistical analyses in subgroups of patients ever treated and never treated as the prediction of incident vertebral fracture and reclassification analysis were not performed because of the low sample size of each subgroup and the very low number of incident fractures.

Our results suggest that FRAX® with BMD performed just as well as the combination of history of fracture, age and FN BMD. However comparing the performance of an internally derived model to an external predictive model provides the best predictive performance because the internally derived model is constructed to best fit the data within the cohort, whereas an external model is necessarily derived from other sources.

FRAX® was not developed to predict incident radiographic vertebral fractures; our analysis is only exploratory and our results should be interpretated cautiously. Finally, our models are conditional on the estimates of the 10-yr probability of fracture developed by FRAX®.

The strengths of our study include the assessment of fracture risk in relevant population i.e. postmenopausal women from a random population without any selection biases. The vertebral fractures were carefully assessed, on standardized X-rays with central analysis. The prospective methodology of our study allows adequate assessment of potential confounders including the main risk factors for fractures. Moreover, we took into account the limitations of the ROC curve methodology, criticized because they are applied to diagnostic criteria and are not appropriate to judge the performance of predictive algorithms [33], [34]. Thus we performed reassignment analysis using net reclassification improvement (NRI) [33], [34] and confirmed that the addition of BMD improved the performance of FRAX® [36]. The reassignment analysis needs arbitrary choice of the fracture threshold; 20% for major osteoporotic fracture is proposed by NOF and is a widely accepted value used to estimate risk model performance. Our study shows that the addition of BMD to FRAX® improves the prediction of patients and could permit the careful selection of patients who should receive the highest priority for treatment, in order to have the better risk-benefit ratio.

In conclusion, we showed that, in a cohort of European postmenopausal women recruited from the general population, FRAX® can identify those at highest risk of incident major osteoporotic fracture and incident radiographic vertebral fracture. Different tests used to evaluate FRAX performance for prediction of major osteoporotic fractures showed that FRAX® with BMD performed better than FRAX without BMD.
